# Datasets on materials research of hard ferromagnet in TM-Fe-Si (TM=Ti, Zr, Hf, V, Nb, and Ta) ternary systems

**DOI:** 10.1016/j.dib.2022.108868

**Published:** 2022-12-30

**Authors:** Ryusei Ishibashi, Jiro Kitagawa

**Affiliations:** Department of Electrical Engineering, Faculty of Engineering, Fukuoka Institute of Technology, 3-30-1 Wajiro-higashi, Higashi-ku, Fukuoka 811-0295, Japan

**Keywords:** Permanent magnet, X-ray diffraction pattern, Microstructure, Magnetization, Coercive field

## Abstract

The datasets presented in this article are related to materials research on hard ferromagnet in TM-Fe-Si (TM=Ti, Zr, Hf, V, Nb, and Ta) ternary systems. The motivation for data collection is based on the research paper entitled “Novel hard magnetic phase with Zr_11.5_Fe_53_Si_35.5_ composition”. The datasets are composed of scanning electron microscope images, X-ray diffraction (XRD) patterns, and magnetization data for TM_7_Fe_52_Si_41_ annealed at 1050 °C. The chemical compositions of constituent phases were determined by an energy dispersive X-ray spectrometer (EDS). The phase analysis was performed using XRD and EDS results. The Curie temperature of each sample was obtained using magnetization data, and the coercive field was determined for hard ferromagnet samples Zr_7_Fe_52_Si_41_ and Hf_7_Fe_52_Si_41_. The datasets would be useful for developing an Fe-based rare-earth-free permanent magnet, which is one of the central issues of materials science.

**Specifications Table**

**Table:** 

Subject	Metals and alloys
Specific subject area	Phase analyses and ferromagnetic properties of Fe-based compounds
Type of data	Image Figure
How the data were acquired	Scanning electron microscope (SEM), energy dispersive X-ray spectrometer (EDS), powder X-ray diffractometer (XRD), vibrating sample magnetometer (VSM)
Data format	Raw Analyzed
Description of data collection	Each sample was prepared by arc melting constituent elements and annealed at 1050 °C for one day in an evacuated quartz tube. SEM and EDS results were obtained for samples with a polished surface. XRD patterns were taken for powdered samples. VSM data were collected using bulk samples between 50 K and 800 K.
Data source location	• Institution: Fukuoka Institute of Technology • City/Town/Region: Fukuoka • Country: Japan
Data accessibility	Data are with the article. The raw data are in the Mendeley Data repository. https://data.mendeley.com/datasets/gp8rkw2k6v/2

## Value of the Data

• The datasets are useful for elucidating the phase relation in TM-Fe-Si (TM=Ti, Zr, Hf, V, Nb, and Ta) ternary diagrams and designing magnetic properties of an Fe-based compound.
• The data would help the researchers working on the field of rare-earth-free magnetic materials to develop a novel permanent magnet.
• The data can be used for simulating the phase diagram of TM-Fe-Si ternary system and creating a strategy of coercive field enhancement of rare-earth-free magnets.

## 1 Objective

Rare-earth-free magnetic materials like Fe-based compounds are good candidates for low-cost magnets. It is beneficial to collect phase analyses and fundamental magnetic property data obtained through materials research on new Fe-based compounds. The datasets of structural characterization and magnetization in Fe-based materials are indispensable for developing a new rare-earth-free permanent magnet.

## 2 Data Description

**Fig. 1:**
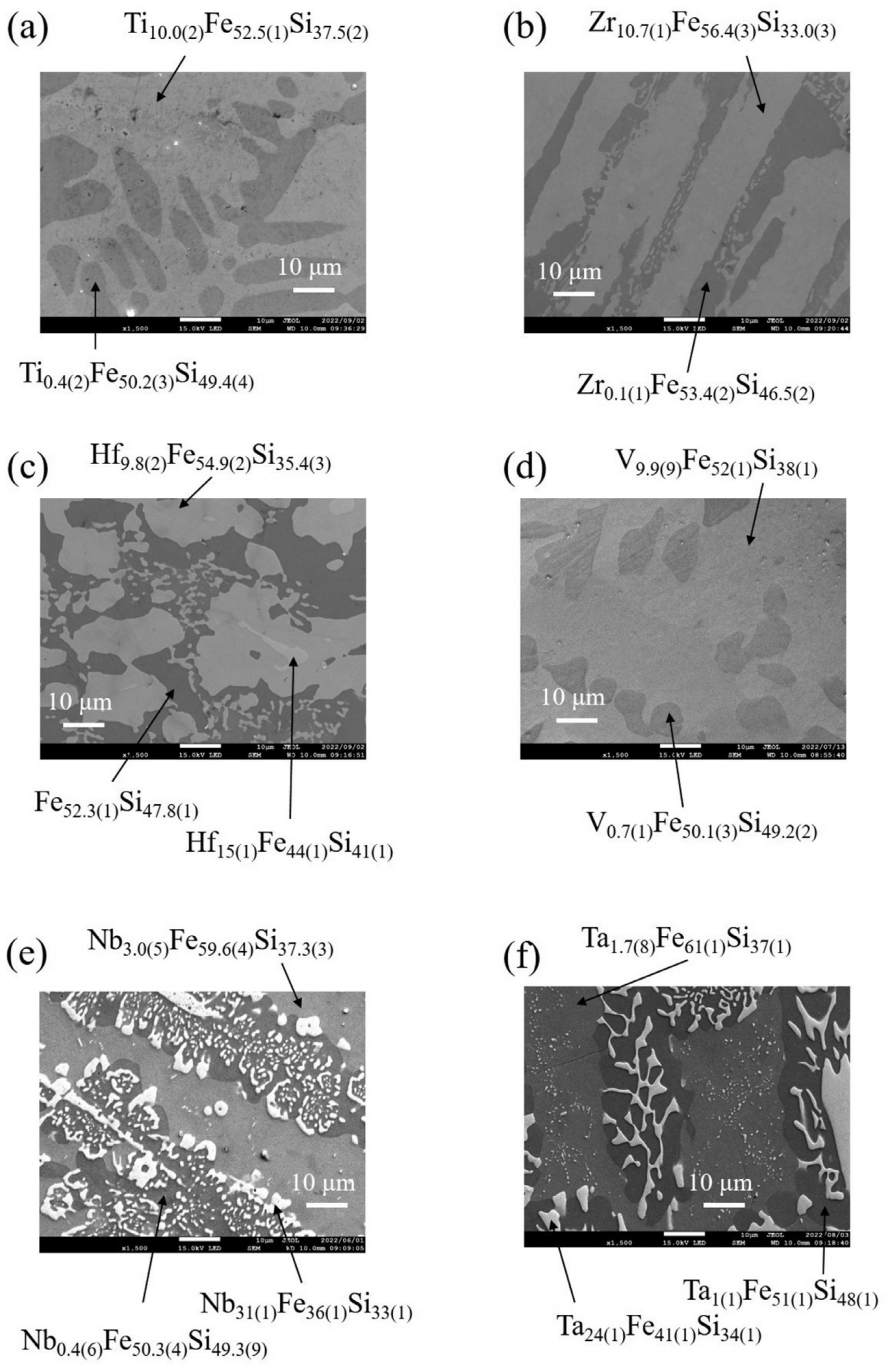
SEM images of (a) Ti_7_Fe_52_Si_41_, (b) Zr_7_Fe_52_Si_41_, (c) Hf_7_Fe_52_Si_41_, (d) V_7_Fe_52_Si_41_, (e) Nb_7_Fe_52_Si_41_, and (f) Ta_7_Fe_52_Si_41_, respectively. In each image, the chemical compositions of phases determined by EDS are also noted.

Recently Yamamoto et al. have discovered a novel hard magnetic phase with Zr_11.5_Fe_53_Si_35.5_ composition [1]. The samples are multiphase, and one of the starting compositions showing superior hard magnetic properties is Zr_7_Fe_52_Si_41_. The microstructure analysis suggests that Zr_7_Fe_52_Si_41_ contains Zr_11.5_Fe_53_Si_35.5_, FeSi, and Fe_5_Si_3_. Motivated by the paper [1], we collected datasets of SEM images, EDS results, XRD patterns, and magnetization to investigate the TM (TM=Ti, Zr, Hf, V, Nb, and Ta) dependences of phase relation and magnetic properties in TM_7_Fe_52_Si_41_. Fig. 1(a) to (f) show the SEM images of TM_7_Fe_52_Si_41_ (TM=Ti, Zr, Hf, V, Nb, and Ta), respectively. Each sample is composed of two or three phases, and the detected chemical compositions are noted in the figure. All samples contain the FeSi phase. In TM=Ti, Zr, and Hf samples, the phases close to the composition of Zr_11.5_Fe_53_Si_35.5_ are detected (Ti_10.0_Fe_52.5_Si_37.5_, Zr_10.7_Fe_56.4_Si_33.0_, Hf_9.8_Fe_54.9_Si_35.4_). The Hf_7_Fe_52_Si_41_ sample exhibits a small amount of Hf_15_Fe_44_Si_41_ with an unknown structure. For TM=V, Nb, and Ta samples, Fe_5_Si_3_ phase with the Mn_5_Si_3_-type structure is observed (V_9.9_Fe_52_Si_38_, Nb_3.0_Fe_59.6_Si_37.3_, and Ta_1.7_Fe_61_Si_37_). Fe_5_Si_3_ might be partially substituted by the TM atom. Nb_7_Fe_52_Si_41_ and Ta_7_Fe_52_Si_41_ samples possess NbFeSi with the TiNiSi-type structure and the C14 Laves phase Ta_24_Fe_41_Si_34_, respectively.

**Fig. 2:**
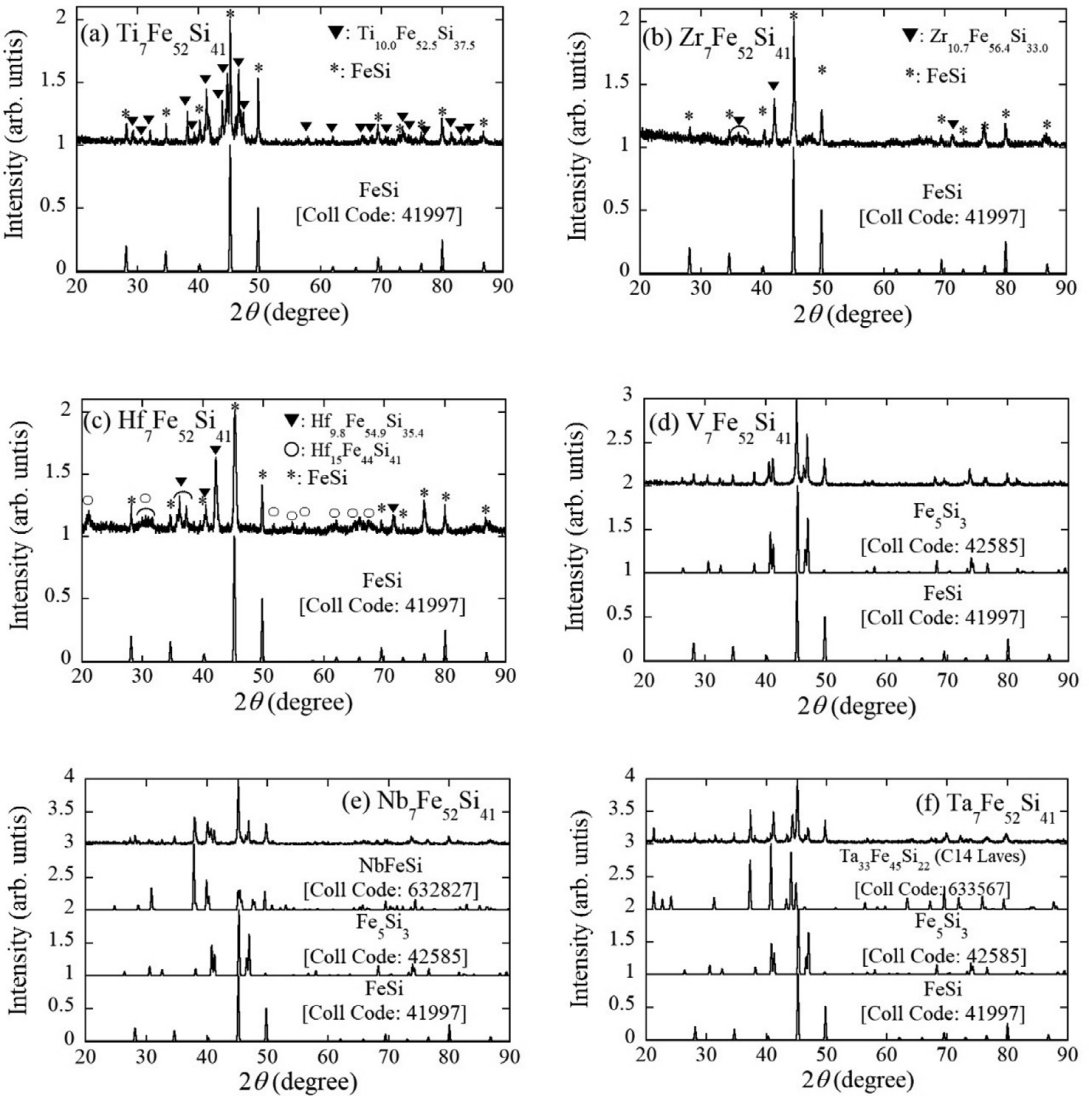
XRD patterns of (a) Ti_7_Fe_52_Si_41_, (b) Zr_7_Fe_52_Si_41_, (c) Hf_7_Fe_52_Si_41_, (d) V_7_Fe_52_Si_41_, (e) Nb_7_Fe_52_Si_41_, and (f) Ta_7_Fe_52_Si_41_, respectively. In each figure, the simulation pattern of the assigned phase with a known crystal structure is added. The origin of each pattern is shifted by an integer value for clarity.

The XRD patterns of TM_7_Fe_52_Si_41_ (TM=Ti, Zr, Hf, V, Nb, and Ta) are presented in Fig. 2(a) to (f). The simulation patterns of some compounds with known crystal structures in the ICSD database are added. The coll code of the ICSD database for each simulation pattern is given in the figure. The peak assignments are carried out taking into account the EDS results (see also Fig. 1(a) to (f)). All samples contain the XRD pattern of FeSi. In the Ti_7_Fe_52_Si_41_ sample, the diffraction peaks other than the FeSi XRD pattern would originate from Ti_10.0_Fe_52.5_Si_37.5_ with an unknown structure (▼ in Fig. 2(a)). Although the crystal structure of Zr_11.5_Fe_53_Si_35.5_ composition is unclear, the paper [1] has reported the XRD pattern of the phase. We also confirmed the XRD pattern of Zr_11.5_Fe_53_Si_35.5_ phase (▼ in Fig. 2(b)). Almost the same pattern is detected in the Hf_7_Fe_52_Si_41_ sample, as shown by ▼ in Fig. 2(c). The diffraction peaks other than FeSi and Hf_9.8_Fe_54.9_Si_35.4_ XRD patterns would be assigned as the Hf_15_Fe_44_Si_41_ phase. We note that the intensity of the reflections at 35-38°attributed to the Zr_11.5_Fe_53_Si_35.5_ phase in Hf_7_Fe_52_Si_41_ is larger compared to Zr_7_Fe_52_Si_41_ (see ▼ in Fig. 2(b) and (c)). The unknown Hf_15_Fe_44_Si_41_ phase may contribute to 35-38°reflections marked by ▼. While the chemical composition of Ti_10.0_Fe_52.5_Si_37.5_ is similar to that of Zr_10.7_Fe_56.4_Si_33.0_ or Hf_9.8_Fe_54.9_Si_35.4_, the XRD pattern of Ti_10.0_Fe_52.5_Si_37.5_ is different from that of Zr_10.7_Fe_56.4_Si_33.0_ or Hf_9.8_Fe_54.9_Si_35.4_. The diffraction peaks of V_7_Fe_52_Si_41_ shown in Fig. 2(d) can be assigned as Fe_5_Si_3_ or FeSi phase. In Nb_7_Fe_52_Si_41_, the diffraction peaks due to NbFeSi with the TiNiSi-type structure appear in addition to the XRD patterns of Fe_5_Si_3_ and FeSi (Fig. 2(e)). As indicated in Fig. 2(f), the XRD pattern of Ta_7_Fe_52_Si_41_ can be explained by the superposition of those of Ta_33_Fe_45_Si_22_ (C14 Laves phase), Fe_5_Si_3_, and FeSi. The chemical composition for the C14 Laves Ta_33_Fe_45_Si_22_ in the simulation patterns is near to Ta_24_Fe_41_Si_34_ detected by EDS (see also Fig. 1(f)). The raw data of experimental XRD patterns are stored in the Mendeley Data repository.

**Fig. 3:**
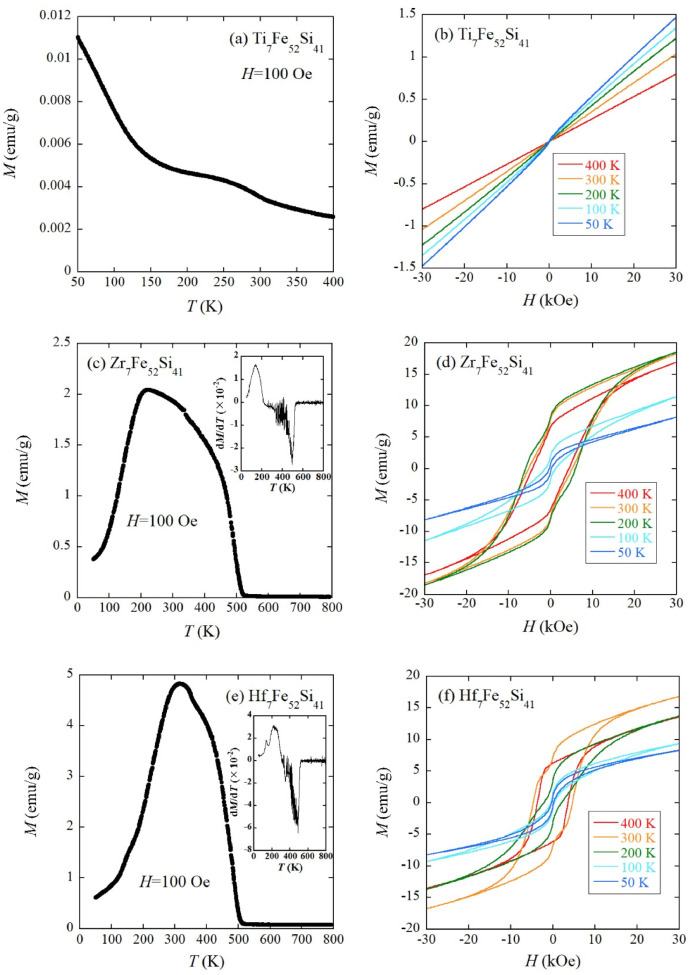
(a) Temperature dependence of *M* and (b) isothermal *M*-*H* curves at 50 K, 100 K, 200 K, 300 K, and 400 K for Ti_7_Fe_52_Si_41_. The same datasets are displayed for Zr_7_Fe_52_Si_41_ ((c) and (d)) and Hf_7_Fe_52_Si_41_ ((e) and (f)). Insets of (c) and (e) are the temperature derivative of *M* for Zr_7_Fe_52_Si_41_ and Hf_7_Fe_52_Si_41_, respectively.

**Fig. 4:**
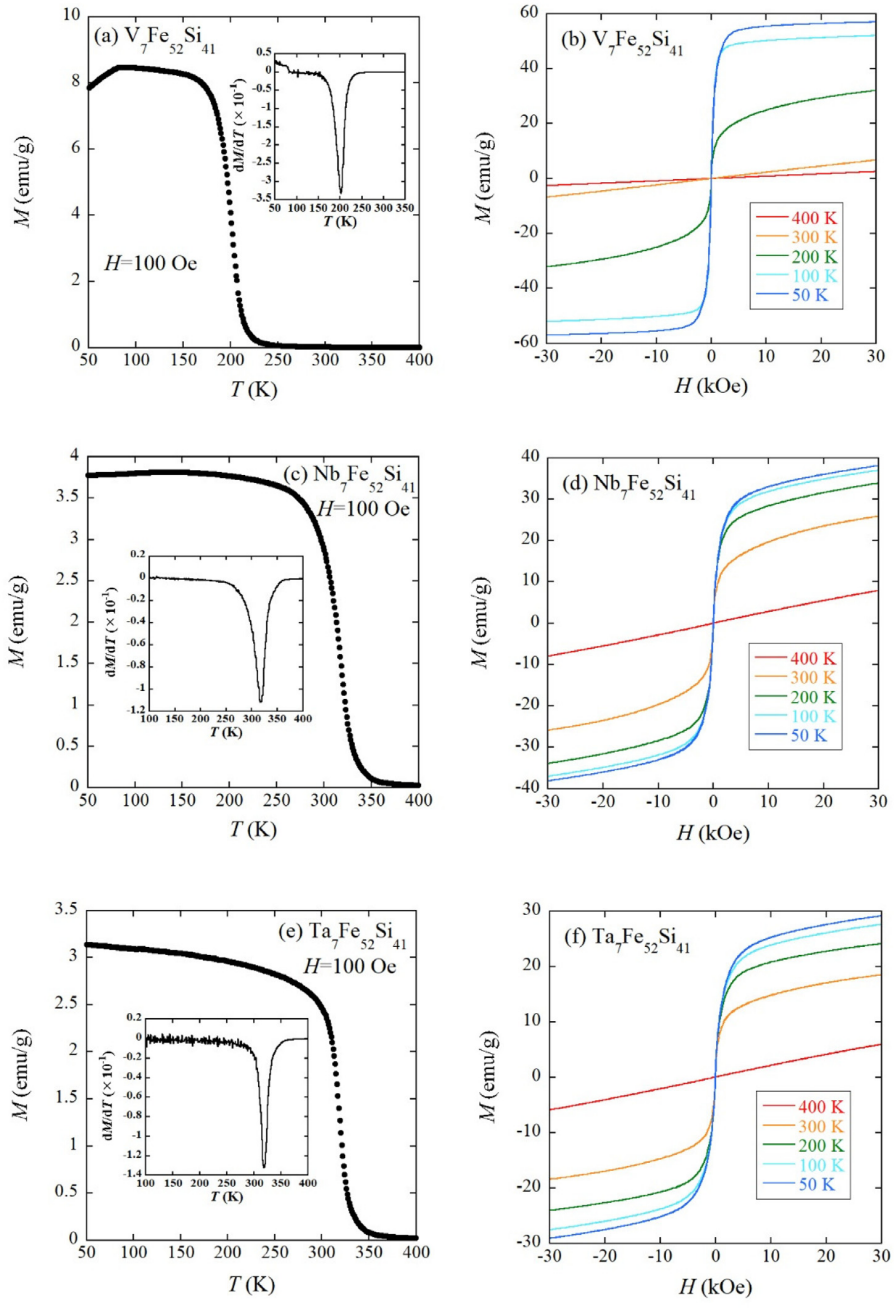
(a) Temperature dependence of *M* and (b) isothermal *M*-*H* curves at 50 K, 100 K, 200 K, 300 K, and 400 K for V_7_Fe_52_Si_41_. The same datasets are displayed for Nb_7_Fe_52_Si_41_ ((c) and (d)) and Ta_7_Fe_52_Si_41_ ((e) and (f)). Insets of (a), (c), and (e) are the temperature derivative of *M* for V_7_Fe_52_Si_41_, Nb_7_Fe_52_Si_41_, and Ta_7_Fe_52_Si_41_, respectively.

Fig. 3(a) to (f) summarize the magnetic property data of TM_7_Fe_52_Si_41_ (TM=Ti, Zr, and Hf). The temperature dependence of dc magnetization *M* was measured under the external field *H* of 100 Oe. Ti_7_Fe_52_Si_41_ exhibits smaller *M* compared to Zr_7_Fe_52_Si_41_ and Hf_7_Fe_52_Si_41_ (Fig. 3(a), (c), and (e)). *M*-*H* (*M*: magnetization) curves of Ti_7_Fe_52_Si_41_ (Fig. 3(b)) showing almost linear behaviors indicate a paramagnetic character at least down to 50 K. Zr_7_Fe_52_Si_41_ and Hf_7_Fe_52_Si_41_ enter into a ferromagnetic state below approximately 500 K (Fig. 3(c) and (e)). *T*_C_ (Curie temperature) values of Zr_7_Fe_52_Si_41_ and Hf_7_Fe_52_Si_41_ are 495 K and 488 K, respectively, which are determined by the minimum point of temperature derivative of *M* (insets of Fig. 3(c) and (e)). This analysis method is commonly employed for transition metal-based ferromagnets [2,3]. *T*_C_ of Zr_7_Fe_52_Si_41_ is close to the reported value (533 K) [1]. Fig. 3(d) and (f) suggest large coercive fields *H*_c_ for Zr_7_Fe_52_Si_41_ and Hf_7_Fe_52_Si_41._ In Zr_7_Fe_52_Si_41_, *H*_c_=5.3 kOe at 300 K is comparable with the reported value (4.2 kOe) [1] and is unchanged down to 200 K. At 100 K and 50 K, we confirmed the reduced *H*_c_. Hf_7_Fe_52_Si_41_ also shows a relatively large *H*_c_ of 4.7 kOe at 300 K; however, *H*_c_ gradually decreases as the temperature is lowered below 300 K. The magnetic property data of TM_7_Fe_52_Si_41_ (TM=V, Nb, and Ta) are presented in Fig. 4(a) to (f). Each sample shows a ferromagnetic transition, as shown in Fig. 4(a), (c), or (e). The insets of Fig. 4(a), (c), and (e) showing the temperature derivative of *M* indicate that *T*_C_ values of V_7_Fe_52_Si_41_, Nb_7_Fe_52_Si_41_, and Ta_7_Fe_52_Si_41_ are 202 K, 318 K, and 318 K, respectively. The *M*-*H* curves exhibit typical soft ferromagnetic character below approximately *T*_C_ (Fig. 4(b), (d), and (f)). The raw data of Fig. 3(a) to (f) and Fig. 4(a) to (f) are stored in the Mendeley Data repository.

## 3 Experimental Design, Materials and Methods

Polycrystalline samples with a mass of 1.5 g were synthesized by a home-made arc furnace using constituent elements Ti (99.9 %), Zr (99.5 %), Hf (98 %), V (99.9 %), Nb (99.9 %), Ta (99.9 %), Fe (99.9 %), and Si (99.999 %) under Ar atmosphere. The atomic ratio was TM (=Ti, Zr, Hf, V, Nb, or Ta): Fe: Si =7: 52: 41. The button-shaped samples were remelted several times on a water-cooled Cu hearth and flipped each time to ensure homogeneity. Each as-cast sample was subsequently placed in an evacuated quartz tube and annealed at 1050 °C for one day, followed by an air-cooling in an electric furnace. Room temperature X-ray diffraction (XRD) patterns of annealed samples were collected using an X-ray diffractometer (XRD-7000L, Shimadzu) with Cu-Kα radiation in Bragg-Brentano geometry. The phase analysis was carried out using a field emission scanning electron microscope (FE-SEM, JSM-7100F, JEOL). After checking the SEM images, the chemical composition in each area (∼ 5μm × 5μm) was measured by an energy dispersive X-ray spectrometer (EDS) equipped with the FE-SEM. The chemical composition was determined by averaging several data collection points. The temperature dependence of dc magnetization *M* (*T*) between 50 and 400 K was measured using a vibrating sample magnetometer (VSM) option in VersaLab (Quantum Design). The high-temperature *M* (*T*) from 400 K to 800 K was measured by another VSM (TM-VSM33483-HGC, Tamakawa). In each measurement, the external field (*H*) was 100 Oe. The isothermal magnetization (*M)* curves between *H*=-30 kOe and *H*=30 kOe at 50 K, 100 K, 200 K, 300 K, and 400 K were measured using the VSM option of VersaLab.

## Ethics Statements

The authors followed universally expected standards for ethical behavior in conducting and publishing scientific research.

## CRediT authorship contribution statement

**Ryusei Ishibashi:** Formal analysis, Investigation. **Jiro Kitagawa:** Conceptualization, Methodology, Formal analysis, Writing – original draft, Writing – review & editing, Supervision.

## Declaration of Competing Interest

The authors declare that they have no known competing financial interests or personal relationships that could have appeared to influence the work reported in this paper.

## Data Availability

Datasets on materials research of hard ferromagnet in TM-Fe-Si (TM=Ti, Zr, Hf, V, Nb, and Ta) ternary systems (Original data) (Mendeley Data).
